# Evaluation of the Local and Peripheral Immune Responses in Patients with Cystic Echinococcosis

**DOI:** 10.3390/pathogens13060477

**Published:** 2024-06-04

**Authors:** Linda Petrone, Saeid Najafi-Fard, Laura Falasca, Settimia Sbarra, Antonella Teggi, Emanuele Nicastri, Lucia Rosalba Grillo, Mirco Burocchi, Giuseppe Maria Ettorre, Alessandra Ludovisi, Daniele Colombo, Franca Del Nonno, Delia Goletti

**Affiliations:** 1 Translational Research Unit, National Institute for Infectious Diseases (INMI) “Lazzaro Spallanzani”—IRCCS, 00149 Rome, Italy; 2 Laboratory of Electron Microscopy, National Institute for Infectious Diseases “Lazzaro Spallanzani”—IRCCS, 00149 Rome, Italy; 3 Department of Infectious and Tropical Diseases, Sant’Andrea Hospital University of Rome “Sapienza”, 00189 Rome, Italy; 4 Clinical Division of Infectious Diseases, National Institute for Infectious Diseases “Lazzaro Spallanzani”—IRCCS, 00149 Rome, Italy; 5 Anatomic Pathology Unit, San Camillo-Forlanini Hospital, 00152 Rome, Italy; 6 Division of General Surgery and Liver Transplantation, San Camillo-Forlanini Hospital, 00152 Rome, Italy; 7 Foodborne and Neglected Parasitoses Unit, Department of Infectious Diseases, Istituto Superiore di Sanità, 00161 Rome, Italy; 8 Pathology Unit, National Institute for Infectious Diseases “Lazzaro Spallanzani”—IRCCS, 00149 Roma, Italy

**Keywords:** cystic echinococcosis, cysts, immune response, immunohistochemistry, IL-4

## Abstract

Background: Cystic echinococcosis (CE) cysts may persist for decades because of immune modulation mechanisms. Here, we characterize the cysts and the blood immune responses in patients with CE. Methods: We enrolled 61 patients with CE and 19 control subjects. We received tissue samples from seven patients with CE and a control subject requiring liver cystectomy. The immunohistochemistry evaluation of the immune cell subtypes and cytokines in the pericysts and surrounding liver and the antigen B (AgB)-specific response analysis of whole blood were performed. Results: In CE, the pericyst and the surrounding liver parenchyma showed aggregates of CD3^+^ T lymphocytes, mainly CD4^+^. B lymphocyte aggregates were present in the liver tissue. Monocytes/granulocytes were rarely observed. Th2 cytokine expression was scarce, whereas IFN-γ expression was present in the CE tissues. The control subject did not show an inflammatory infiltrate. The IL-4-specific response to AgB was increased in the patients with CE compared to the control, and this result was confirmed in a larger cohort (*p* = 0.003), whereas the IFN-γ-response was similar between the two groups (*p* = 0.5570). Conclusion: In patients with CE, CD4^+^ lymphocytes infiltrate the pericyst and the surrounding liver tissue with a low IL-4/IL-13 expression level and a moderate IFN-γ expression level; moreover, an IL-4 parasite-specific response is detected in the periphery. These results support adventitia involvement in CE immunopathogenesis.

## 1. Introduction

Cystic echinococcosis (CE) is a zoonotic disease caused by *Echinococcus granulosus* sensu lato. Humans are accidental intermediate hosts and become infected by ingesting echinococcal eggs, which develop into fluid-filled cysts in tissues, mainly the liver. An echinococcal cyst has a complex structure; the lumen of the cyst, filled with the so-called hydatid fluid, is surrounded by a parasite component, the germinal, and the laminated layers. The outer part of the cyst is a host-derived adventitia layer. The germinal layer is the thin inner part of the cyst composed of embryonic cells that generate the protoscoleces (PSC) and the laminated layer. The laminated layer is an a-cellular structure composed by highly glycosylated glycoproteins whose role is to maintain the physical integrity of the cyst and to protect the germinal layer from the host’s immunity [1,2]. It has been demonstrated that immune cells can interact both with the outer surface of the laminated layer and with the soluble excretory/secretory products of the parasite. Therefore, for this reason, the laminated layer may be considered an interface between the parasite and the host [1], playing an important role in parasite immune evasion and immune modulation. The outer layer of the echinococcal cyst, i.e., the adventitia or pericyst, is a fibrous layer mainly consisting of epithelial cells and connective tissue that limit and protect the cyst. This layer is generated by the host cellular inflammatory response and show the infiltration of eosinophils, fibroblasts, and mesothelial cells [3]. However, the immune mechanisms that lead to the formation of the adventitia have not been completely characterized.

In an intermediate host, untreated echinococcal cysts, even evolving in terms of size, morphology characteristics, and viability, may persist for decades. This indicates that the parasite develops several mechanisms of evasion from the host protective response or that there is immune modulation trying to contain the parasite’s growth over time. In this context, besides the laminated layer, the pericyst may be also involved in local immune regulation during cyst establishment and growth. Indeed, the pericyst reaction to the parasite is supposed to contribute to cyst infertility, as infertile bovine cysts have shown disorganization of the laminated layer depending on the adventitia infiltration of host macrophages, thus suggesting a potential role for these cells in determining the fate of the viability of the cyst [4]. Moreover, live cysts show a scarce inflammatory reaction, whereas a relevant granulomatous reaction was observed in bovine regressive cysts [1,5]. Furthermore, experimental studies in in vivo models demonstrated that livers at the cyst establishment phase show an immune cell infiltrate of both T (with a T1, T2, or Treg phenotype) and B lymphocytes; natural killer (NK) and NKT cells are also involved [6]. Moreover, mice with dead cysts show pericyst-infiltrating leucocytes producing high levels of both Th1 and Th2 cytokines, whereas mice with live cysts showed high levels of IL-10 [7,8]. Cells expressing forkhead box (Fox) P3 or regulatory cytokines, such as Interleukin (IL)-10 or Transforming Growth Factor (TGF)-β, have been recently identified in tissues from sheep with CE [9,10]. All these data suggest a role of the adventitia layer in the immunopathogenesis of CE. 

The current knowledge on immune responses in human CE derives mainly from studies on whole blood cells or serological assays, which are all in agreement with the coexistence of Th1 and Th2 responses [11,12,13]. This immune profile may have an impact also on the tissue immune response during chronic CE. Studies on cyst evolution in humans are complex; nonetheless, the evaluation of cyst cellular infiltrates and the functional capacities of these cells and how the cellular dynamics impact cyst growth may help in addressing this issue [14]. So far, little is known about the local immune response to human cysts. Few studies investigated local immune cell infiltration, demonstrating a larger T cell population in the host tissue reaction site of human echinococcal cysts [12,15] and reporting a prevalence of CD4 T cells or CD8 CTL cells [12,15], depending on the tissue evaluated. Nuclear factor kappa B (NF-κB), a well-known immune regulator, and inducible nitric oxide synthase (iNOS) were over-expressed in patients with primary infection, whereas the inhibition of the NF-κB/iNOS pathway was found in recurrent CE, thus suggesting an important role of these factors in host protection [16]. Also, B and T regulatory cells have been recently investigated [15]. Finally, Th1-type and Th17-type RNA profiles were found in the pericyst of echinococcal cysts from patients with inactive cysts, whereas a Th2 profile was prevalent in tissues from patients with active cysts [17]. 

In this study, we aimed to concomitantly characterize both the local immune and the peripheric immune responses in patients with cystic echinococcosis. The immunohistochemistry evaluation of the immune cell subtypes in the pericysts and surrounding liver as well as the antigen-specific immune response analysis of whole blood were performed.

## 2. Material and Methods

### 2.1. Study Population

Subjects suspected of CE followed at the outpatient clinic of Istituto Nazionale per le Malattie Infettive (INMI) “Lazzaro Spallanzani”-IRCCS (Rome, Italy) at Sant’Andrea Hospital (Rome, Italy) and at the Division of General Surgery and Liver Transplantation, POIT Department, San Camillo Hospital-“Lazzaro Spallanzani” National Institute for Infectious Diseases (INMI)-IRCCS (Rome, Italy), who signed the informed consent form for research purposes were prospectively enrolled in the study. Among them, the subjects requiring cystectomy were characterized for local and peripheral immune responses. Pre-surgery blood samples and tissue samples from surgically removed human echinococcal cysts were requested. This study was approved by the Ethics Committees of all the institutions involved in this study (approval numbers: 28/2014, 59/2014, 16/2018, 146/2020, 34/2010, and 436/11).

The CE cysts were diagnosed based on the presence of pathognomonic features via ultrasound or other radiological techniques depending on cyst localization [18]; based on the cysts and clinical characteristics, the best treatment option was chosen. The cysts from patients requiring cystectomy were staged according to the WHO-Informal Working Group on Echinococcosis (WHO-IWGE) classification; therefore, the CE stage was available for removed liver cysts as the classification was applied during diagnosis and prior to surgery [17,19,20,21]. The WHO-IWGE classification identifies six stages (CE1, CE2, CE3a, CE3b, CE4, and CE5) based on the pathognomonic features of cysts, characterizing the active–transitional–inactive ultrasonographic status of the cyst. Moreover, based on the metabolic profiles of cysts, it is possible to include in the active (and viable) group the CE1, CE2, and CE3b cysts. The “transitional” group includes the CE3a stage, as CE3a cysts have equal probability to be viable and non-viable, while the “inactive” group includes the CE4 and CE5 cysts [22]. To simplify group classification, patients with more than one cyst at different stages were classified according to the more active stage [20,23,24,25]. Routine serology tests performed at INMI were the NovaLisa Echinococcus IgG ELISA (NovaTec Immunodiagnostica GmbH, Dietzenbach, Germany) and the Western Blot IgG (Euroimmun Labordiagnostika, Luebeck, Germany). 

The diagnosis of CE is based on imaging techniques, and serology tests can support diagnosis, where imaging is inconclusive [18,19,26,27]. Therefore, the clinical inclusion criterion for patients with CE was the presence of ≥1 CE cyst at any stage and in any organ. The clinical inclusion criterion for the controls was the presence of non-parasitic focal lesions in the liver or other organs that could lead to the differential diagnosis of CE (e.g., biliary cysts and neoplasms). Clinically relevant controls were included as these subjects may have had an inflammatory infiltrate not correlated to *E. granulosus*, thus allowing for the study of cellular infiltrates in patients with CE in a more specific manner. Age < 18 years was an exclusion criterion for both the CE and control groups. Demographic and clinical information were collected at the time of enrolment. 

### 2.2. Immunohistochemistry

Tissue samples comprising the adventitia layer and surrounding liver were rinsed with Hanks Balanced Salt Solution (HBSS) media (Sigma Aldrich-Merck, Darmstadt, Germania), kept in 10% natural buffered formalin, and then paraffin-embedded. Four micron-thick sections were cut from paraffin blocks, stained with hematoxylin and eosin, or with antibodies specific for lymphocytes, monocytes, and granulocytes or cytokines. For immunohistochemistry, the sections were immersed in 10 mM sodium citrate, pH 6.0, and microwaved for antigen retrieval and stained using the BenchMark ULTRA system fully automated instrument (Roche/Ventana BenchMark XT; Roche Diagnostics, Indianapolis, IN, USA) with an antibody directed against CD3 rabbit (Roche Diagnostics, 2GV6) CD4 rabbit (Roche Diagnostics, SP35), CD8 rabbit (Roche Diagnostics, SP57), CD14 rabbit (Roche Diagnostics, EPR3653), CD20 mouse (Roche Diagnostics, L26), CD23 rabbit (Roche Diagnostics, SP23), CD15 mouse (Roche Diagnostics, MMA), IL-4 rabbit (Boster Biological Technology, Pleasanton, CA, USA; PA1749), IL-13 rabbit (Boster Biological Technology, Pleasanton, CA, USA; RP1047), IL-5 rat (Biolegend, San Diego, CA, USA; JES1-39D10), and Interferon (IFN)-γ rabbit (Abcam, Cambridge, UK; ab9657). The use of an antibody diluent instead of the primary antibody in the immunohistochemical procedure served as the negative control.

All cases were independently analyzed by two pathologists, without knowledge of clinical diagnosis. The extent of immunoreactivity was assessed by using the same microscope at a magnification of 40×. The proportions of positive cells were scored and classified into 4 groups (score 1: 0%; score 2: <10%; score 3: 10–30%; score 4: >30%). For CD15^+^ and for IFN-γ cells, the following scores were applied: score 1: 0%; score 2: 1–5%; score 3: >5<20%; score 4: >20<50%; or score 5: >50%. 

### 2.3. Whole Blood Test

Five-six hundred microliters of heparinized whole blood was stimulated with 1–4 µg/mL of native AgB-enriched fraction [28,29,30] and incubated overnight at 37 °C with 5% CO_2_ as previously described [28,29,30]. The supernatants were then harvested and frozen until cytokine analysis. Unstimulated whole blood and blood stimulated with 200 ng/mL staphylococcal enterotoxin B (Sigma Aldrich) served as the negative and positive controls, respectively. 

### 2.4. Cytokine Analysis

The Ag-specific response was evaluated by measuring IL-4 level using highly sensitive ELISA (Quantikine HS IL-4 ELISA, R&D Systems, Minneapolis, MN, USA), or measuring the IFN-γ level using an ELISA assay routinely used in our lab (www.quantiferon.com) according to the manufacturer’s instructions. The IL-4 range of detection was 0.25-16 pg/mL. The IFN-γ limit of detection was 0.065 IU/mL. Sera/plasma from surgically treated patients were also tested for immune/growth factors [IL-1β, IL-1RA, IL-2, IL-4, IL-5, IL-6, IL-7, IL-8, IL-9, IL-10, IL-12p70, IL-13, IL-15, IL-17A, eotaxin, basic fibroblast growth factor (FGF), granulocyte colony-stimulating factor (G-CSF), granulocyte-macrophage colony-stimulating factor (GM-CSF), IFN-γ, IFN-γ induced protein 10 (IP-10), monocyte chemoattractant protein 1 (MCP-1), macrophage inflammatory protein (MIP)-1α, MIP-1β, platelet-derived growth factor (PDGF), RANTES (regulated on activation, normal T cell expressed and secreted), tumor necrosis factor-α (TNF-α), and vascular endothelial growth factor (VEGF)] using Bio-Plex Pro Human Cytokine 27-plex Assay panel and the Bioplex200 system (all from Bio-Rad, Hercules, CA, USA) following the manufacturer’s instructions. The data were analyzed as previously described [31,32,33]. All the stimulated samples were subtracted from the unstimulated control. For the unstimulated samples, the level of each soluble factor was analyzed based on the standard curve range of production.

### 2.5. Statistical Analysis

Prism 8 software (Graphpad Software 8.0, San Diego, MO, USA) was used for data graphing and statistic procedures. Medians and interquartile ranges (IQRs) were calculated for continuous measures; the data were analyzed using non-parametric tests when appropriate. *p* values were considered significant if *p* ≤ 0.05.

## 3. Results

### 3.1. Population Characteristics

We enrolled a total of 61 patients with CE and 19 control subjects during the 2013–2016 period. The clinical and demographical characteristics are reported in Table 1a. Within this cohort, we received tissue samples from seven patients with CE and a control subject requiring liver cystectomy (Table 1b). The subjects described in Table 1b were studied in parallel for local immune and peripheric immune responses. Six patients with CE had active CE2/CE3b cysts, whereas one in seven had a CE3a cyst. Most of the patients (6/7, 85.7%) had liver cysts only, whereas a patient had two liver cysts, one of which required cystectomy, and a lung cyst. The control subject had a serous cyst in the liver with sediment and a blood clot. Part of this study population has previously been studied [28,29,30].

### 3.2. Lymphocytes CD4^+^ Infiltrate the Pericyst and the Surrounding Liver Tissue

**Figure 1:**
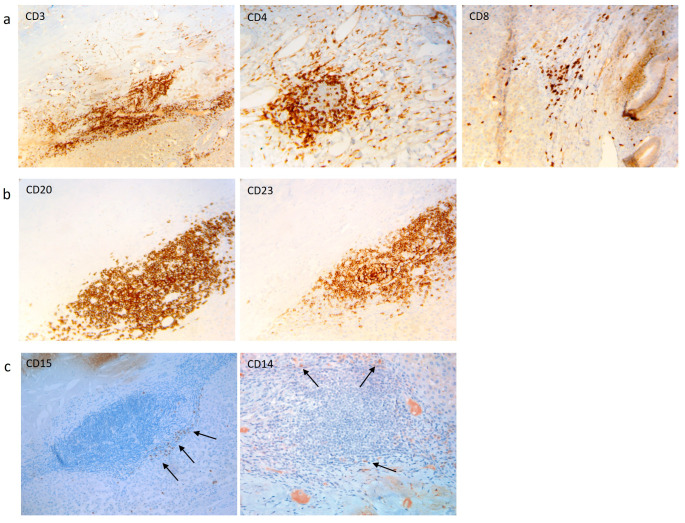
Representative images of the immunohistochemical evaluation of immune cell subsets. (**a**) T lymphocytes; (**b**) B lymphocytes; (**c**) neutrophils (**left** panel) and monocytes/macrophages (**right** panel). The arrows indicate the cells stained with the respective marker. The arrows are included only for images where the staining of a given marker is not widespread, but restricted to the well-identified cells. Original magnification: 20×.

To study local immunity to echinococcal cysts in the human patients with CE, we first evaluated the immune cell distribution in the pericyst and in the surrounding tissue of the liver infected with hydatid cysts. To compare the inflammatory responses, analysis was also performed on the subject with non-echinococcal cysts. As previously demonstrated [15], in the pericyst and even more in the liver parenchyma, aggregates of CD3^+^ T lymphocytes were observed (Table 2 and Figure 1a).

The CD4^+^ T lymphocytes were better represented compared to the CD8^+^ T cells (Table 2 and Figure 1a). B lymphocytes were present in the liver tissue only as follicular aggregates (Table 2 and Figure 1b). Monocytes/macrophages CD14^+^ and granulocytes CD15^+^ were rarely present (Table 2 and Figure 1c). When classifying the patients based on the cyst stage, no specific differences were observed between the patients with active CE2/CE3b cysts and the patients with a CE3a cyst. The evaluation of the absolute numbers of the same cellular type, referred to as the whole blood count collected on the day of surgery or a few days before, did not show modulations (Supplementary Table S1). The only control subject evaluated did not show an inflammatory infiltrate (Table 2).

### 3.3. An E. granulosus-Specific Response Was Detected in the Periphery but Not at the Site of the Host Inflammatory Reaction

**Figure 2:**
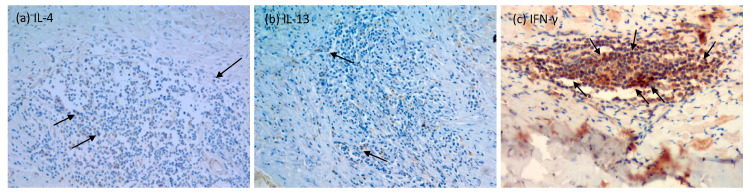
Representative images of the immunohistochemical evaluation of Th2 and Th1 cytokines in the pericyst. (**a**) IL-4 immunolabelling; (**b**) IL-13 immunolabelling; (**c**) IFN-γ immunolabeling. The arrows indicate the cells stained with the respective marker. Original magnification: 20×.

Considering the presence of T lymphocytes at the site of the host cyst reaction, we wondered whether these cells are functionally active. Therefore, we evaluated the expression of Th2 cytokines, IL-4, and IL-13 and of the IFN-γ, the main Th1 cytokine. As shown in Table 3 and Figure 2a,b, Th2 cytokines were scarce or absent in both the pericyst and liver parenchyma of the patients with CE, whereas a moderate expression of IFN-γ (Figure 2c) was present in both the pericyst and the surrounding liver of the patients with CE. 

**Figure 3:**
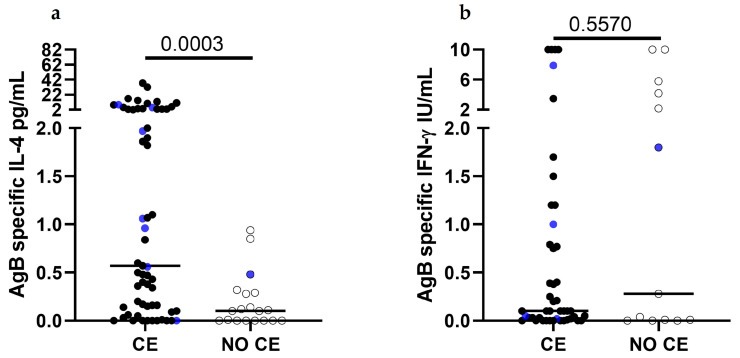
Increased IL-4 response to AgB is associated with CE. (**a**) The IL-4 levels are increased in the patients with CE (black dots) compared to those of the control (empty dot) (*p* = 0.0003) enrolled during the 2013–2016 period. The same trend was observed for the patients with CE and controls who required cystectomy (blue dots); (**b**) the IFN-γ levels are similar in the patients with CE (black dots) and controls (empty dots) (*p* = 0.5570) enrolled during the 2013–2016 period. The same trend was observed for the patients with CE and controls who required cystectomy (blue dots). Footnotes: The horizontal bars represent medians. The IL-4 and IFN-γ concentrations were determined by ELISA; the IFN-γ response was available for 46/61 patients with CE and 13/19 controls. The responses were compared using the Mann–Whitney test; the differences were considered significant at *p*-values of ≤0.05.

The levels of 27 different chemokines, cytokines, and growth factors in the unstimulated plasma of the surgically treated patients were also low or absent (Supplementary Table S2). The only chemokine that showed a high level of production was RANTES (median 7113 pg/mL, IQR: 4487 pg/mL–10,721; standard curve range: 15,709–0.96 pg/mL). In contrast, the analysis of IL-4 after whole blood stimulation with an enriched-AgB fraction showed an increased IL-4 level in the patients with CE compared to that of the control subject (Supplementary Figure S1a). The same analysis performed on a larger population of patients with CE and controls showed that the IL-4-specific response is significantly associated with CE (*p* = 0.0003, Figure 3a), as previously demonstrated [28,29]. We also analyzed IFN-γ production in response to the enriched-AgB fraction. Unfortunately, the data available for a part of the surgically treated patients (3/8) (Supplementary Figure S1b) showed no evident trends of response; however, the same analysis performed on a larger population (46/61 patients with CE and 13/19 controls) showed that the IFN-γ-specific response is similar between the patients with CE and the controls (*p* = 0.5570, Figure 3b).

## 4. Discussion

Echinococcal cysts may persist in intermediate hosts for decades due to the establishment of mechanisms of immune modulation or immune evasion. The adventitia is described as the host’s reaction to the parasite with the infiltration of several immune cell types. Moreover, several studies demonstrated the coexistence of both Th1 and Th2 responses in patients with CE, depending on the cyst stage [11,13]. These responses may certainly impact the cellular composition and the immune responses at the cyst level. Therefore, in this study, we aimed to concomitantly evaluate both the local immune and the peripheric immune responses of patients with CE requiring liver cystectomy. At the local level, we found aggregates of CD3^+^ T lymphocytes, mainly CD4^+^ T lymphocytes in the pericyst and the surrounding liver parenchyma. B lymphocytes aggregates were present, mostly in the liver tissue. Monocytes and granulocytes were rarely observed in both pericyst and liver parenchyma, as well as Th2 cytokine expression, which was scarce. In contrast, IFN-γ was observed in both the pericyst and surrounding liver.

At the blood level, the IL-4 antigen B-specific response was increased in the patients with CE compared to the control, and this result was significantly confirmed in a larger cohort.

The cell composition of the adventitia and their functionality in human cysts was evaluated in a few studies, demonstrating the presence of B lymphocytes, rare polymorphonuclear cells, and monocytes in fertile cysts [12,15]. Consistently, we found a few granulocytes and monocytes. However, even if B cells were not the most represented cells in our sample, they were organized in aggregates, as previously demonstrated [15]. T lymphocytes were the most abundant cells found in the pericysts of human and non-human cysts [5,12,15]. Moreover, we found that CD4 T cells were more represented compared to CD8 T cells, as previously demonstrated [12,15]. Moreover, CD8 T cells were the most represented population in cattle progressive cysts, whereas CD4 T cells are associated with regressive cysts [5,9], thus suggesting that the lymphocytes that are locally activated may have an impact on cyst evolution. However, in our setting, there were no differences in CD4 or CD8 densities between the active CE2/CE3b and the transitional CE3a cysts. The cellular composition of the surrounding liver mirrored the adventia infiltrates, showing, in some cases, a greater cellular density mainly for T lymphocytes, thus suggesting the active recruitment of T lymphocytes around the parasite, with the aim of controlling cyst growth. Unfortunately, due to staining problems, we could not evaluate if the CD4 T cells abundantly represented in our samples were regulatory T cells.

Besides the characterization of cellular infiltrates in human pericysts, it is also important to understand if these cells can produce cytokines. Here, we evaluated Th2 and Th1 cytokines in the pericyst and in the surrounding liver by immunohistochemistry; we did not find the expression of IL-4 or IL-13, while the expression of IFN-γ was present in both the pericyst and the liver parenchyma, suggesting the prevalence of the Th1 response, at least in the few cases analyzed in our setting. However, the low number of patients with CE classified within each group hampered our effort to evaluate the correlation between immune response polarization and the viability of the cysts. In this regard, it has been demonstrated that Th1-type and Th17-type RNA profiles were found in the patients with inactive cysts, whereas a Th2 profile was prevalent in the tissues from patients with active cysts [17], thus suggesting that the host immune response may be different depending on the cyst stages. However, besides the important value of putting more pieces in the puzzle of CE immunopathogenesis, the clinical usefulness of these findings is a matter of debate, considering that liver biopsy is unfeasible for diagnostic or follow-up purposes. In parallel, the evaluation of 27 different chemokines, cytokines, and growth factors in the unstimulated plasma of the surgically treated patients showed a fair level of production of RANTES, a chemokine involved in the recruitment of leucocytes to the site of inflammation. However, the low number of subjects evaluated and the absence of a consistent number of subjects in the control population did not allow us to depict a clear picture of the circulating immune factors. On the other hand, the ex vivo assessment of Th1 and Th2 cytokines in the sera from the patients with CE and controls did not show important differences between the groups, and this is not surprising as peripheral cytokines are not antigen-specific [1]. In contrast, although the low number of subjects evaluated hampered us from performing statistical analysis, the assessment of the enriched-AgB fraction-specific response in the whole blood showed an increased IL-4 level in patients with CE compared to the control subject. The same analysis performed in a larger population of patients with CE and controls showed that the IL-4-specific response is significantly associated with CE, as previously demonstrated [28,29]. Similarly, the analysis of the IFN-γ levels showed that the specific IFN-γ response does not associate with CE. Again, the numbers for surgically treated patients were too low to evaluate specific correlations. However, we previously demonstrated that the presence of IL-2+TNF-α+Th2+ triple-positive and TNF-α+Th2+ double-positive specific T cells is associated with biological cyst activity [11], suggesting that a mixed Th1-Th2 response is more present in the periphery in patients with active cysts compared to patients with inactive cysts.

The evaluation of the cellular infiltrates and the functional capacities of these cells may contribute to a better understanding of immune modulations and the regulatory mechanisms underlying the cysts’ evolution. All the studies so far demonstrate that *E. granulosus* and its antigens profoundly alter the architecture and the cellular dynamics across the cyst-harboring tissue. However, the clinical relevance of these findings needs to be addressed in larger CE populations, possibly including the all the cyst stages that may require cystectomy [34] and cysts in different organ locations.

The limitations of this study should be mentioned. First, the evaluation of a low number of patients with CE and controls requiring cystectomy did not allow us to perform any statistical analysis or to analyze the cellular distribution at the site of infection based on the cyst stage. However, since surgery is performed only in a subgroup of patients with CE [18], it is expected to enroll patients with CE with the same cyst stages in our small cohort. Moreover, the stratification of the few patients based on cyst stage showed no specific differences between the patients with active CE2/CE3b cysts and the patient with a CE3a cyst regarding both the cellular distribution and the cytokine production at the site of infection. Another important limit of this study was the lack of parasite genotype evaluation as an important factor influencing organ tropism, and therefore as a potential factor in modulating the host immune response. A recent systemic review demonstrated that *E. granulosus* s.s. (G1, G3) is responsible for the majority of CE human infection in Europe [35]. Therefore, our cases may be likely driven by *E. granulosus* s.s. Further studies are needed to better address this issue. Besides the genotype, additional studies may be useful to understand how the tissue harboring the cyst may impact the host’s local response. Indeed, the low number of subjects evaluated in this study hampered our efforts in assessing if cysts in locations different from the liver have a different cellular infiltrate or cytokine profile. In addition, the evaluation of the cytokine profiles of the patients with CE and controls with a different sample size is arguable; however, the inclusion of clinically relevant controls is demanding, mainly in the case of complex diseases such as cystic echinococcosis. Finally, we evaluated a low number of cytokines at the site of infection; this was due to the technical difficulties related to antigen retrieval; further studies involving different methodologies, such as mRNA-level evaluation, as previously employed in other studies [12,17], will be useful to better characterize the immune profile of cells infiltrating the pericyst and the surrounding tissue.

In conclusion, our study expands the knowledge on the cellular composition of the adventitia and of the surrounding liver during CE. We found that CD4^+^ lymphocytes infiltrate the pericyst and the surrounding liver tissue with a low expression level of Th2 cytokines and a moderate expression level of the Th1 cytokine IFN-γ; moreover, a Th2 parasite-specific response was detected in the periphery. These results support the role of the adventitia layer in the immunopathogenesis of CE.

## Figures and Tables

**Table 1:** (**a**) Demographical and clinical characteristics of enrolled patients in 2013–2016. (**b**) Demographical and clinical characteristics of the subjects analyzed at local and blood levels.

(a)	CE		No CE	*p* Value
N (%)	61		19	
Median Age year (IQR) *	55 (41–71)		52 (49–69)	0.74
**Female gender N (%)**	30 (49.2)		10 (52.6)	0.79
**Serology positive results N (%)**	41 (67.2)		0 (0)	**<0.0001**
**Previous pharmacological treatment**	26 (42.6)		NA	
**Current pharmacological treatment**	19 (31.1)		NA	
Cyst number/patient median (IQR) **	1 (1–2)		1 (1–2) ***	0.18
**(b)**	**CE**		**No CE**	
	**CE2/CE3b**	**CE3a**		
**N (%)**	**6 (85.7)**	**1 (14.3)**	**1 (100.0)**	
**Median Age year (IQR)**	53 (39–69)	30 (NA)	**50** (NA)	
**Female gender N (%)**	2 (66.6)	1 (100.0)	1 (100.0)	
**Serology positive results N (%)**	6 (100.0)	0 (0)	0 (0)	
**Previous pharmacological treatment**	4 (66.7)	0 (0)	NA	
**Cyst number/patient median (IQR)**	2 (1–3)	2 (NA)	2 (NA)	

Footnotes: N: number; IQR: interquartile range; * available in 60/61 patients with CE ** subjects with multiple cysts were excluded; *** info missing for 2 subjects. NA: not applicable.

**Table 2:** Scoring of immune cells in the pericyst and surrounding liver.

		CE				NO CE	
		CE2/CE3b		CE3a			
Type of Cell	% of Positive Cells Evaluated	Pericyst	Liver	Pericyst	Liver	Pericyst	Liver
Lymphocytes CD3^+^ N (%)	0	0 (0)	0 (0)	0 (0)	0 (0)	1 (100)	0 (0)
	<10	2 (33.3)	0 (0)	1 (100)	0 (0)	0 (0)	1 (100)
	10–30	2 (33.3)	1 (16.7)	0 (0)	0 (0)	0 (0)	0 (0)
	>30	2 (33.3)	5 (83.3)	0 (0)	1 (100)	0 (0)	0 (0)
Lymphocytes CD4^+^ N (%)	0	0 (0)	0 (0)	0 (0)	0 (0)	1 (100)	0 (0)
	<10	0 (0)	0 (0)	1 (100)	0 (0)	0 (0)	1 (100)
	10–30	5 (83.3)	1 (16.7)	0 (0)	1 (100)	0 (0)	0 (0)
	>30	1 (16.7)	5 (83.3)	0 (0)	0 (0)	0 (0)	0 (0)
Lymphocytes CD8^+^ N (%)	0	0 (0)	0 (0)	0 (0)	0 (0)	1 (100)	0 (0)
	<10	5 (83.3)	2 (33.3)	1 (100)	0 (0)	0 (0)	1 (100)
	10–30	1 (16.7)	3 (50.0)	0 (0)	1 (100)	0 (0)	0 (0)
	>30	0 (0)	1 (16.7)	0 (0)	0 (0)	0 (0)	0 (0)
Lymphocytes CD20^+^ N (%)	0	3 (50.0)	0 (0)	0 (0)	0 (0)	1 (100)	0 (0)
	<10	3 (50.0)	4 (66.7)	0 (0)	0 (0)	0 (0)	1 (100)
	10–30	0 (0)	2 (33.3)	0 (0)	1 (100)	0 (0)	0 (0)
	>30	0 (0)	0 (0)	1 (100)	0 (0)	0 (0)	0 (0)
Lymphocytes CD23^+^ N (%)	0	6 (100.0)	4 (66.7)	1 (100)	0 (0)	1 (100)	1 (100)
	<10	0 (0)	1 (16.7)	0 (0)	1 (100)	0 (0)	0 (0)
	10–30	0 (0)	1 (16.7)	0 (0)	0 (0)	0 (0)	0 (0)
	>30	0 (0)	0 (0)	0 (0)	0 (0)	0 (0)	0 (0)
Monocytes CD14^+^ N (%)	0	1 (16.7)	0 (0)	1 (100)	0 (0)	0 (0)	0 (0)
	<10	2 (33.3)	3 (50.0)	0 (0)	1 (100)	1 (100)	1 (100)
	10–30	1 (16.7)	1 (16.7)	0 (0)	0 (0)	0 (0)	0 (0)
	>30	2 (33.3)	2 (33.3)	0 (0)	0 (0)	0 (0)	0 (0)
Granulocytes CD15^+^ N (%)	0	2 (33.3)	0 (0)	0 (0)	0 (0)	1 (100)	0 (0)
	1–5	3 (50.0)	2 (33.3)	0 (0)	0 (0)	0 (0)	1 (100)
	>5<20	0 (0)	2 (33.3)	1 (100)	0 (0)	0 (0)	0 (0)
	>20<50	1 (16.7)	1 (16.7)	0 (0)	0 (0)	0 (0)	0 (0)
	>50	0 (0)	1 (16.7)	0 (0)	1 (100)	0 (0)	0 (0)

Footnotes: N: number of cases; CE: cystic echinococcosis; CD: cluster of differentiation.

**Table 3:** Scoring of Th2 cytokines in the pericyst and surrounding liver.

		CE				NO CE	
		CE2/CE3b		CE3a			
Type of Cytokine	% of Positive Cells Evaluated *	Pericyst	Liver	Pericyst	Liver	Pericyst	Liver
IL-4 N (%)	0	4 (66.7)	2 (33.3)	1 (100)	1 (100)	1 (100)	0 (0)
	<10	2 (33.3)	4 (66.7)	0 (0)	0 (0)	0 (0)	0 (0)
	10–30	0 (0)	0 (0)	0 (0)	0 (0)	0 (0)	1 (100)
	>30	0 (0)	0 (0)	0 (0)	0 (0)	0 (0)	0 (0)
IL-13 N (%)	0	5 (83.3)	2(33.3)	0 (0)	0 (0)	1 (100)	0 (0)
	<10	1 (16.7)	2 (33.3)	1 (100)	0 (0)	0 (0)	0 (0)
	10–30	0 (0)	2 (33.3)	0 (0)	1 (100)	0 (0)	1 (100)
	>30	0 (0)	0 (0)	0 (0)	0 (0)	0 (0)	0 (0)
IFN-γ (%) *	0	0 (0)	0 (0)	0 (0)	0 (0)	-	-
	1–5	0 (0)	0 (0)	1 (100)	0 (0)	-	-
	>5 < 20	3 (100)	2 (66.7)	0 (0)	1 (100)	-	-
	>20 < 50	0 (0)	1 (33.3)	0 (0)	0 (0)	-	-
	>50	0 (0)	0 (0)	0 (0)	0 (0)	-	-

Footnotes: N: number of cases; CE: cystic echinococcosis; * IFN-γ staining was available in 4/8 subjects.

## Data Availability

The raw data generated and/or analyzed within the present study will be available in the INMI institutional repository (rawdata.inmi.it), subject to registration. The data can be found by selecting the article of interest from a list of articles ordered by year of publication. No charge for granting access to the data is required. In the event of the malfunction of the application, the request can be sent directly by e-mail to biblioteca@inmi.it.

## Supplementary Materials

The following supporting information can be downloaded at: https://www.mdpi.com/article/10.3390/pathogens13060477/s1, Table S1: Absolute counts of peripheral circulating cells in patients with CE; Table S2: Plasma level of cytokines, chemokines, and growth factors evaluated in surgically treated patients with CE; Supplementary Figure S1: IL-4 and IFN-γ responses to AgB in patients with CE and controls requiring cystectomy.
