# Supplementary material for: Evaluation of the Local and Peripheral Immune Responses in Patients with Cystic Echinococcosis
Source: Pathogens. 2024 Jun 4;13(6):477. doi: 10.3390/pathogens13060477 (PMC11206897; doi:10.3390/pathogens13060477)
Supplement: Supplementary file 1 [file pathogens-13-00477-s001.zip › pathogens-2957251-supplementary.pdf]

Table S1. **Absolute counts of peripheral circulating cells in patients with CE**

	CE		NO CE
	**CE2/CE3b**	**CE3a**	
**Type of cell** median (IQR) x10^3 µl	N=6	**N=1**	**N=1**
Lymphocytes	1.7 (0.9-2.6)	0.6	1.7
Monocytes	1.1 (0.7-1.8)	0.8	0.4
Neutrophils	11.3 (5.9-17.6)	13.3	4.6
Eosinophils	0.05 (0-0.3)	0	0

Abbreviations: CE: cystic echinococcosis; IQR: interquartile range.

Table S2. Plasma level of cytokines, chemokines, and growth factors evaluated in surgically treated patients with CE*

Function	Main Source	Analyte	Median pg/mL (IQR)
Pro-Inflammatory cytokines/chemokines	Activated macrophages	**IL-1β**	1.2 (0.92-1.49)
	Macrophages	**IL-6**	0.28 (0-1.8)
	Macrophages	**IL-8**	26.84 (20.88-78.18)
	Th9	**IL-9**	371.2 (304.4-435.5)
	Th17	**IL-17A**	17.86 (16.43-24.13)
	Th1	**IFN-γ**	8.2 (6.56-18.65)
	Monocytes, Fibroblasts, Endothelial Cells	**IP-10**	252.1 (205.2-271.5)
	Monocytes, Macrophages	**MCP-1**	62.20 (42.88-65.41)
	Th2	**Eotaxin**	79.56 (49.82-124.3)
	Monocytes/Macrophages	**MIP-1α**	2.72 (1.90-4.65)
	Monocytes/Macrophages	**MIP-1β**	140.0 (124.8-156.6)
	Platelets, Macrophages	**RANTES**	7113 (4487-10721)
	Macrophages	**TNF-α**	136.7 (108.5-157.1)
Anti-inflammatory cytokines	Th2	**IL-4**	1.84 (1.32-2.86)
	Neutrophils, Monocyte/Macrophages	**IL1ra**	515.9 (237.1-2101)
	Th2, Treg	**IL-10**	1.62 (0.09-4.08)
	Th2	**IL-13**	0.8 (0.6-1.1)
Growth factors	Th1	**IL-2**	3.24 (1.64-3.43)
	Th2, Mast cells	**IL-5**	6.84 (0.0-16.38)
	DCs	**IL-7**	0 (0-7)
	DCs	**IL-12**	18.58 (4.64-23.04)
	Macrophages	**IL-15**	0 (0-30.56)
	Stromal cells, Macrophages	**FGF-basic**	12.08 (9.12-13.77)
	endothelium, macrophages,	**G-CSF**	130.5 (59.19-248.4)
	macrophages, T cells, mast cells, NK cells	**GM-CSF**	0.96 (0.57-1.74)
	Platelets, Macrophages	**VEGF**	198.6 (125.9-228.1)
	Platelets	**PDGF**	677.8 (506.5-856.3)

Footnotes: CE: cystic echinococcosis; Th: T helper; DCs: dendritic cells; NK: natural killer; IL: interleukin; IFN: interferon; MCP: monocyte chemoattractant protein; MIP: macrophage inflammatory protein; IP: Interferon gamma-induced protein; RANTES: regulated on activation, normal T cell expressed and secreted; TNF: tumour necrosis factor; FGF: fibroblast growth factor; G-CSF: granulocyte-colony stimulating factor; GM-CSF: granulocyte-macrophage colony-stimulating factor; VEGF: vascular endothelial growth factor; PDGF: platelet-derived growth factor; IQR: interquartile range. *samples were available for 6/7 CE patients.

Supplementary Figure S1

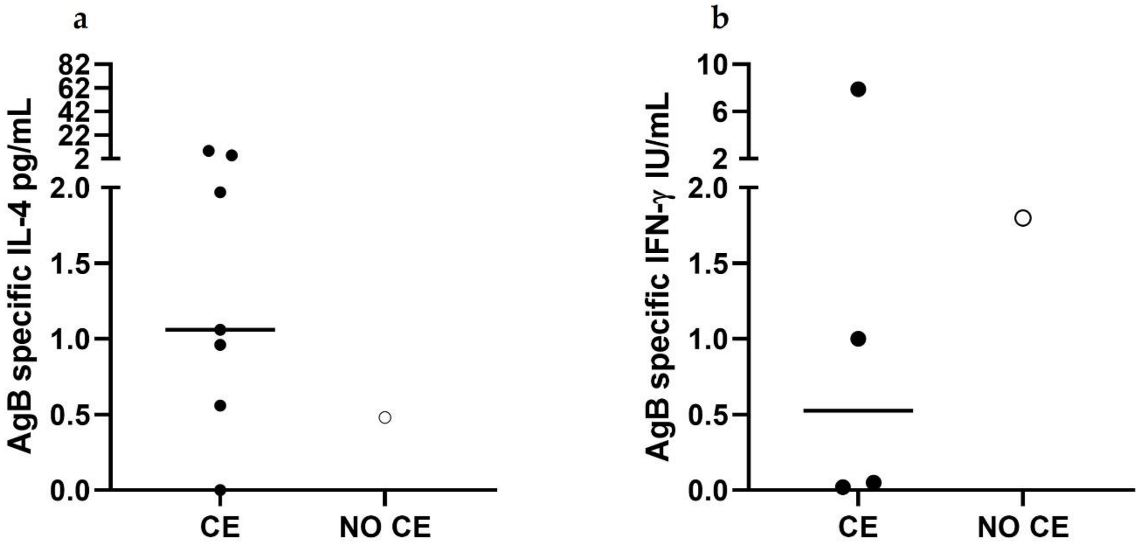

Supplementary Figure S1. Increased IL-4 response to AgB is associated with CE. (a) IL-4 levels are increased in CE patients (black dots) compared to the control (empty dot) who requiring cystectomy; (b) IFN-γ levels in CE patients (black dots) and in the control (empty dot) who required cystectomy. **Footnotes**: Horizontal bars represent medians. IL-4 and IFN-γ concentrations were determined by ELISA; IFN-γ response was available for 4/7 CE patients.
